# Combining Thermal Desorption with Selected Ion Flow Tube Mass Spectrometry for Analyses of Breath Volatile Organic Compounds

**DOI:** 10.1021/acs.analchem.3c04286

**Published:** 2024-01-20

**Authors:** Ilaria Belluomo, Sophia E. Whitlock, Antonis Myridakis, Aaron G. Parker, Valerio Converso, Mark J. Perkins, Vaughan S. Langford, Patrik Španěl, George B. Hanna

**Affiliations:** ⊥ Department of Surgery and Cancer, Imperial College London, London W12 0HS, United Kingdom; ‡ Syft Technologies Limited, 68 St. Asaph Street, Christchurch 8011, New Zealand; § Element Lab Solutions, Wellbrook Court, Girton Road, Cambridge CB3 0NA, United Kingdom; ∥ J. Heyrovský Institute of Physical Chemistry of the Czech Academy of Sciences, 182 23 Prague, Czechia

## Abstract

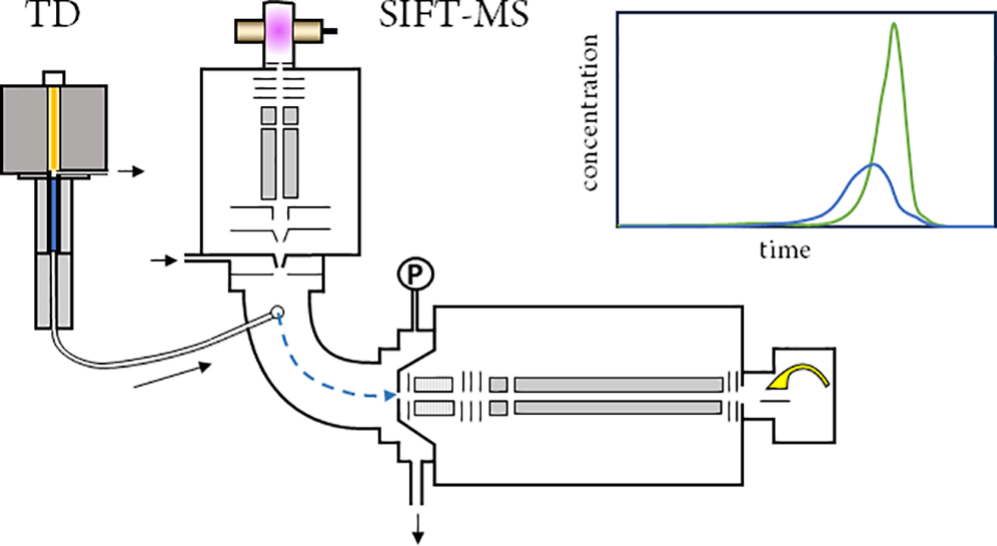

An instrument integrating thermal desorption (TD) to selected ion flow tube mass spectrometry (SIFT-MS) is presented, and its application to analyze volatile organic compounds (VOCs) in human breath is demonstrated for the first time. The rationale behind this development is the need to analyze breath samples in large-scale multicenter clinical projects involving thousands of patients recruited in different hospitals. Following adapted guidelines for validating analytical techniques, we developed and validated a targeted analytical method for 21 compounds of diverse chemical class, chosen for their clinical and biological relevance. Validation has been carried out by two independent laboratories, using calibration standards and real breath samples from healthy volunteers. The merging of SIFT-MS and TD integrates the rapid analytical capabilities of SIFT-MS with the capacity to collect breath samples across multiple hospitals. Thanks to these features, the novel instrument has the potential to be easily employed in clinical practice.

Volatile organic compound (VOC) analysis within exhaled breath represents an attractive noninvasive strategy for diagnosis and therapeutic monitoring. VOCs emitted by the body reflect biochemical processes underlying physio-pathological states.^1^ VOCs produced by both normal and irregular metabolism within human cells and gut bacteria may travel within systemic circulation before being released by the lungs.^2,3^ Alterations of breath profiles have been reported in different diseases,^4^ including different types of cancers^5,6^ and respiratory diseases.^7^ Breath tests are noninvasive and therefore well-accepted by patients, representing an adequate and affordable method to assess subjects with nonspecific symptoms. In a recent study, 1002 adult patients were recruited in primary care to test the acceptability and feasibility of the breath test; 98% of the recruited subjects found the test to be acceptable and easy to perform.^8^ In addition, the wide applicability of breath analysis has been further proved by the high acceptability in infants and children.^9^

Despite all the advantages, breath is a complex biological matrix. Many VOCs have structural similarities and are present at low concentrations; therefore, the techniques for their analyses need to be sensitive and specific. Mass spectrometry provides high sensitivity and the possibility to identify compounds with a degree of confidence. The instruments used to analyze VOCs in breath are typically either chromatographic, with gas chromatography mass spectrometry (GC-MS) being the current gold standard, or direct sampling. Direct sampling instruments, among which one widely used for breath analysis is selected ion flow tube mass spectrometry (SIFT-MS), offer the advantage of real-time results and direct quantification.^10^ Patients can directly breathe into the inlet of the instrument, without the requirement for breath collection and storage.^10^ Real-time results are displayed during the analysis, which usually lasts around 1 min. However, SIFT-MS instruments in their current form are not well-suited to large-scale multicenter clinical studies, where thousands of patients are recruited, often simultaneously in different hospitals. Given the nature of analysis, the instrument would need to be located where the recruitment takes place and multicenter studies are not possible to perform. When analyzed with GC-MS, breath is collected in thermal desorption (TD) tubes containing a sorbent that has the capacity to capture VOCs. TD tubes are stored, transported, and later analyzed by a TD unit coupled to a GC-MS instrument, usually with automated methods. TD tubes are an ideal tool for clinical studies, since they are robust and easy to transport and store. In addition, breath collected onto TD tubes can remain stable for a long time.^11,12^ However, the coupling with GC-MS results in slow analysis time due to the time required for efficient chromatographic separation.

The coupling of SIFT-MS with TD for VOC measurement in breath is an attractive analytical approach. The run time per sample can be drastically reduced compared to a chromatographic instrument. Development of tailored methods for specific projects can allow many samples to be run in a short time. Validated TD-SIFT-MS methods could be used in the future for high-throughput screening, in a complementary approach with GC-MS that offers the possibility of a deep untargeted analysis.^13^ To date, relatively few studies have been published that describe the coupling of TD units and SIFT-MS.^14−16^ However, previous work did not achieve the development of a reliable, high-throughput, and effective method that can be used in the clinical environment.

In this study we describe a novel coupling of SIFT-MS with TD. For the first time, TD and SIFT-MS interfaces have been integrated to create a novel hybrid instrument for the measurement of VOCs in human breath, with the potential to be employed in large-scale clinical projects. We developed and validated a targeted analytical method for 21 compounds, chosen for their clinical and biological relevance, following adapted European Medical Agency (EMA) guidelines for the validation of analytical techniques.^17^ Validation has been carried out by two independent teams, the Hanna Group at the Department of Surgery and Cancer, Imperial College London in the United Kingdom and the Syft Technologies laboratory in New Zealand.

## Experimental Section

The experiments were carried out using a SIFT-MS instrument integrated with a thermal desorption unit and autosampler. One type of TD tube was used to optimize the method using chemical standards and real breath samples as detailed below.

### SIFT-MS Instrument

The method was developed and validated using a SIFT-MS instrument (Voice200ultra model; Syft Technologies, Christchurch, New Zealand) with helium carrier gas,^1^ connected to a TD 3.5+ thermal desorption unit and CIS4 UPC plus cooled injector system/transfer line (Gerstel GmbH, Mülheim an der Ruhr, Germany) with nitrogen carrier gas, and coupled with a MultiPurpose Sampler Robotic Pro autosampler (Gerstel GmbH, Mülheim an der Ruhr, Germany). SIFT-MS analysis is based on soft chemical ionization by selected reagent ions (H_3_O^+^, NO^+^, O_2_^+^) interacting with sample molecules. Reagent ions are produced in a microwave discharge, selected by a quadrupole mass filter, and injected into a flow of helium through the flow tube. A continuous flow of sample is admixed, and the analyte molecules react with reagent ions producing characteristic product ions. The knowledge of reaction rate constants allows the calculation of compound concentrations.^18,19^ Performance of the Voice200ultra instrument is routinely optimized using the built-in validation system.^1^

### TD Tubes

Biomonitoring TD tubes with a double-bed sorbent phase composed of Tenax TA/Carbograph 5TD (p/n C2-CXXX-5149; Markes International, Llantrisant, UK) were used, providing wide coverage in terms of compound type captured. The tubes were cleaned using a TC20 conditioning station (Markes International, Llantrisant, UK) following manufacturer’s recommendations (2 h, 310 °C, 100 mL/min nitrogen flow).

### Chemical Standards

Twenty-one compounds were included in the method: acetic acid, acetone, benzaldehyde, butanal, butanoic acid, cyclohexane, decanal, dodecane, hexanal, hexanoic acid, isoprene, nonanal, nonanol, octanal, pentanoic acid, phenol, propanal, propanoic acid, toluene, tridecane, and undecanal. Their molecular formulas and relevant SIFT-MS reagent and product ions are listed in Table S1. The biological relevance of these compounds has been reviewed previously.^20^ All analytical standards were purchased from Sigma (Sigma-Aldrich, USA), except for nonanal (Tokyo Chemical Industry, UK). Four stock solutions, one for each chemical class, were made up in methanol and freshly prepared every month. All analyte concentrations in the stock solutions were 0.025 M, except for acetic acid, isoprene, and acetone, reflecting their higher physiological breath concentrations. Working mix (250, 500, 2000, and 100 ppbv for acetic acid, isoprene, acetone and all the other VOCs respectively, considering 500 mL of breath) were made from stock solutions via serial dilution and freshly prepared every week, as well as calibration curve (CC) mix (concentrations reported in Table S2). A 1 μL sample of each CC solution was spiked onto tubes using a Calibration Solution Loading Rig (CSLR, Markes International, Llantrisant, UK). Nitrogen gas was applied to dry purge excess methanol. Methanol was included in the SIFT-MS method as a quality check, to monitor its quantity and potential effect on reagent ion depletion, and to validate dry purging effectiveness. Additionally, 1-propanol, ethanol, and ammonia were monitored in each sample run for quality control purposes. 1-Propanol and ethanol are small alcohols that may occur in ambient air at very high concentrations (ppmv) in hospital environments. Ammonia is also present at high concentrations in ambient air.^21^ It is good practice to monitor their levels to ensure they do not cause depletion of reagent ions, which leads to inaccurate quantification of measured compounds. Standards of these compounds were not included in the mix since quantification was not performed.

### Breath Samples

500 mL of breath was collected from healthy volunteers (REC approval: 17/WA/0161). Breath was collected through a single expiration in a Nalophan bag, connected to a breath collection system. Breath was then transferred onto two TD tubes simultaneously using a flow rate of 200 mL/min for 2.5 min, to pass a defined volume of 500 mL via each tube.

### Data Analysis and Method Validation

Data acquisition was performed using the Maestro software version 1.5.4.23 (Gerstel GmbH, Mülheim an der Ruhr, Germany) and LabSyft software Pro version 1.8.1 (Syft Technologies, Christchurch, New Zealand). Data processing, further analysis, and graphical representation was performed using LabSyft and GraphPad Prism software version 9 (GraphPad software Inc., San Diego, USA). Linear regression was used to construct CCs. Limit of detection (LOD) and limit of quantification (LOQ) were calculated as the 3σ and 10σ uncertainty of the zero calibration (using the following formula LOD = (3·STEYX)/SLOPE; LOQ = (10·STEYX)/SLOPE).^22^ The matrix effect was evaluated by direct slope comparison of identical CC built using chemical standards, with and without adding breath or water. The calibration range was calculated evaluating the residuals of each CC point (accepted between 80% and 120%). Method accuracy was achieved by analyzing five replicates of five calibration levels (Cal 2.5, 5, 10, 50, and 100) on three consecutive days. Accuracy was calculated as percentage of recovery and accepted when higher than 85% and lower than 115%, for all the levels except the LOQ, where it was accepted when between 80% and 120%. Precision was estimated through calculation of intra-assay and inter-assay coefficient of variation (CV%). The numerical value obtained was considered acceptable when lower than 15%. Carryover was evaluated analyzing five empty clean TD tubes after a run of the higher CC point.

## Results and Discussion

### A Novel Instrument Interface

**Figure 1:**
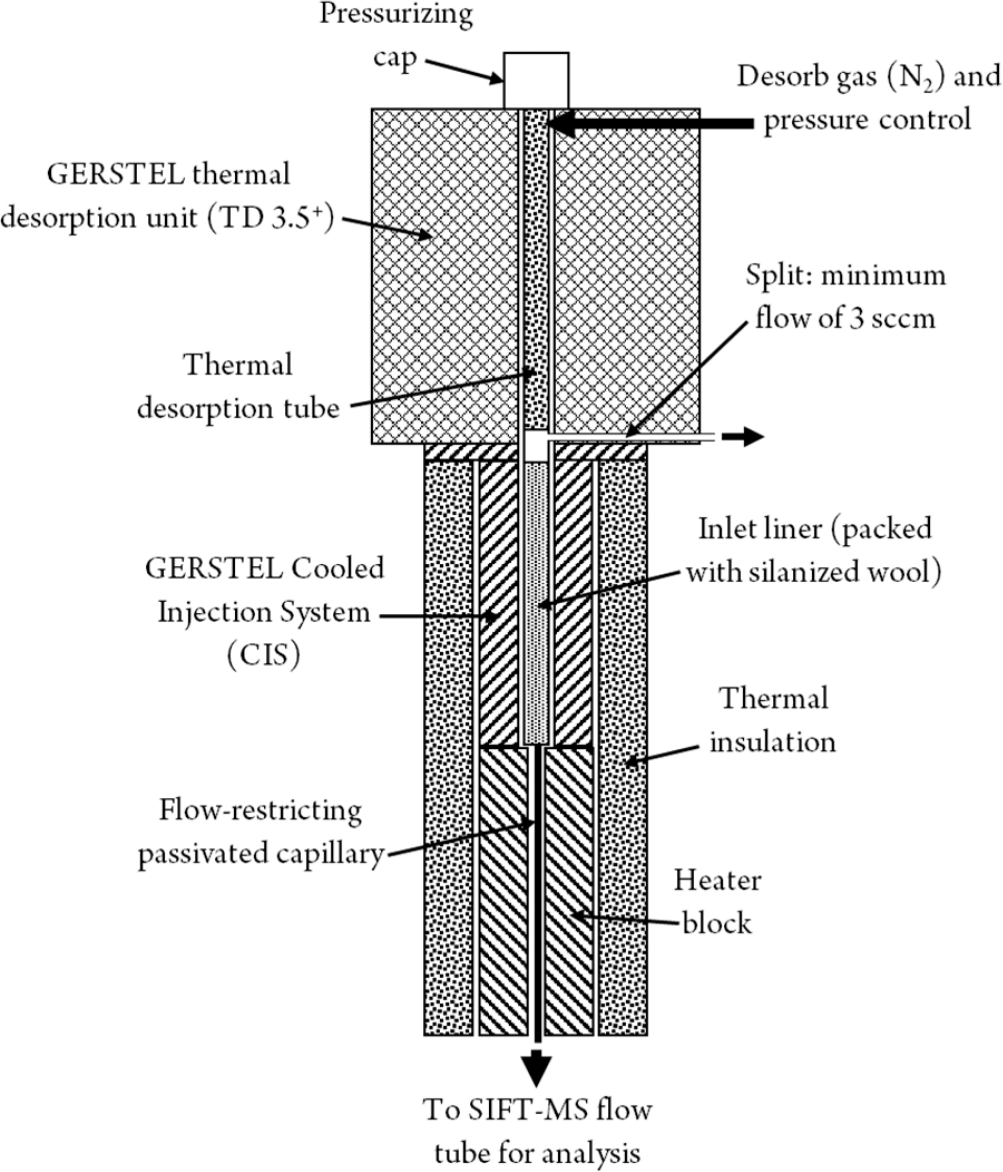
Novel interface designed for the coupling of TD and SIFT.

Breath TD tube analysis was achieved using a novel system, developed collaboratively by GERSTEL and Syft Technologies. This new system enables real-time analysis of desorbed volatiles from TD tubes eliminating the need for timely chromatographic separation. The thermal desorption system, consisting of a TD 3.5+ thermal desorption unit (TDU, Gerstel GmbH, Mülheim an der Ruhr, Germany), was connected to the CIS4 cooled injection system, which operated as a heated transfer line but was retained in the system to facilitate pressure control using the ePneumatics Controller (EPC, Gerstel GmbH, Mülheim an der Ruhr, Germany). This assembly, together with a heater housing, sits atop the SIFT-MS instrument, and the TDU/CIS sample line connects to the inlet capillary, which provides a constant flow rate (25 standard cubic centimeters per minute (sccm)) into the instrument for analysis.^16^ For a complete design of interface, see Figure 1. A standard CIS liner packed with silanized glass wool (Crawford Scientific, Scotland, UK; OD 3 mm (ID 2 mm), length 78 mm) was used in the CIS, to protect the inlet capillary from blockage due to potential contaminating particles (e.g., dust from sorbent tubes). The ePneumatics controller stabilizes and monitors both the desorption gas flow going through the TD tube and the pressure at the downstream end of the TD tube, at the point where the flow splits between the SIFT-MS inlet and the split excess exhaust. The SIFT-MS inlet flow can be adjusted by changing the pressure to reach a desired split-ratio. At both sites, automation of analysis was achieved using an MPS autosampler, enabling unattended analysis with a high throughput. The perfect integration of the different parts made online TD-SIFT-MS sample analysis possible, by mimicking the original direct system typical of the SIFT-MS technique. Further, due to the humidity robustness of SIFT-MS instruments with helium carrier gas, it was not necessary to prepurge breath samples prior to analysis, as is typical for TD-GC analyses. This minimizes the loss of analyte during purging, enhancing the comparability between TD- and direct SIFT-MS.

### Method Development

**Figure 2:**
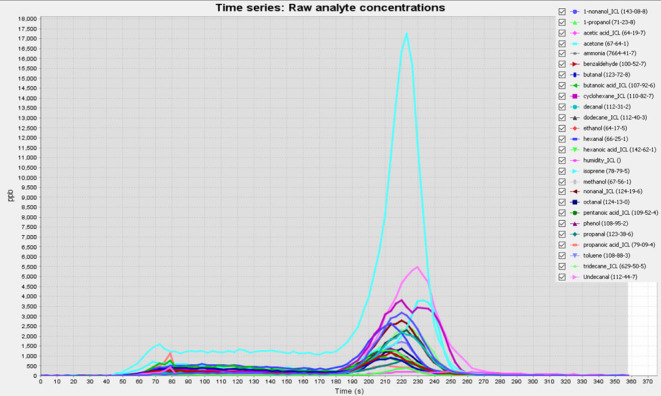
Raw desorption profile of the 21 compounds included in the analytical method, the five compounds monitored for quality purpose and humidity.

Twenty-one compounds of diverse chemical class (acetic acid, acetone, benzaldehyde, butanal, butanoic acid, cyclohexane, decanal, dodecane, hexanal, hexanoic acid, isoprene, nonanal, nonanol, octanal, pentanoic acid, phenol, propanal, propanoic acid, toluene, tridecane, and undecanal), chosen for their biological relevance and their role assessed in previous clinical studies,^5,23^ were targeted in the TD-SIFT-MS analyses. All the compounds included in the analytical method with formula, reagent ion, reaction rate, branching ratio and product ion are summarized in Table S1. Product ion conflicts were resolved using different reagent ions. Different nitrogen flows and purging times were tested to eliminate the excess of methanol. The best values in terms of lower methanol content and analyte loss were obtained using a flow of nitrogen of 135 mL/min through the TD tube for 3 min after standard spiking. This process is necessary for TD tubes spiked with standards dissolved in methanol, but it was not applied for breath samples, since there is no need to dry purge. SIFT-MS is immune to the effects of the water vapor present in breath, and humidity measurement can be used as an additional quality control for adequate breath sampling.^1^ The TD-SIFT-MS analytical method was developed to optimize time resolution of the desorption profile measurement, while enabling the maximum number of analytes to be targeted. Figure 2 shows an example desorption profile of the working mix, containing all compounds included in the method. TD parameters were also optimized. Three desorption temperatures were tested using constant initial temperature, temperature ramp, and a minimum split ratio. The optimal desorption temperature was assessed to be 260 °C. Three values were tested also for the temperature ramp rate, with 160 °C/min giving the best results in terms of analyte desorption profile. Split ratio was optimized to find the best value assuring good sensitivity and avoiding instrument overload. After testing different split ratios, 0.2:1 was chosen. This value represents the minimum value allowed by the Maestro software, assuring minimal compound loss. TD optimized parameters used for the method are summarized in Table 1.

**Table 1:** Final Desorption Parameters Optimized

**Desorption Temperature (°C)**	260
**Temperature Ramp (°C per min)**	160
**Hold Time (min)**	3
**Split Ratio**	0.2:1
**Transfer Line Temperature (°C)**	200
**Standby Temperature (°C)**	50

### Method Validation

Validation of the analytical method was performed following the guidelines on bioanalytical method validation provided by the EMA,^17^ adapted to breath analysis. The validation process was carried out in parallel at the Department of Surgery and Cancer of Imperial College London, London, UK (ICL) and at the Syft Technologies Laboratory, Christchurch, New Zealand (Syft). To overcome the absence of a surrogate matrix, with all breath biological characteristics but without compounds of interest, we used authentic breath from healthy volunteers and water matrices during the validation. Linearity and matrix effect were tested for all the compounds spiking CC pure standards on TD tubes only (triplicates), in combination with water (1 μL, Milli-Q, in duplicates) or breath from healthy volunteers (500 mL, in duplicates). Endogenous content of targeted analytes was subtracted from all the breath CC points. Slopes, intercepts, linear regression coefficient (*R*^2^), calibration range, LOD, and LOQ found by each laboratory carrying out the validation are listed for each compound and matrix in Table S3. Acetic acid, acetone, and isoprene had a higher calibration range compared to all other compounds, due to their higher physiological concentrations in breath, and this is reflected in the LOD and LOQ calculated values. For acetone and isoprene, it was not possible to determine a precise calibration range, LOD, and LOQ in breath matrix since the content of these two compounds in the breath obtained from volunteers and used to build the CC was too high and “masked” the spiked standard. The CCs were consistent in terms of slopes for all the compounds across the three biological matrices for both ICL and Syft data sets (figure S1). Accuracy was calculated between 85% and 115% of the theoretical value for all compounds, at all five calibration levels in both laboratories, with few exceptions. Similar results were obtained for the precision, intraday, and interday measurements. All CV% results were lower than the established threshold of 15%, except for a few cases (higher calculated value outside acceptance limit: 22%, intraday precision for propanal lower level at Syft). The measured levels with standard deviation, accuracy, CV% intraday, and CV% interday are presented in Table S4. These data showcase the good repeatability of the method on different days and concentration ranges. Carryover was also tested by analyzing five empty conditioned TD tubes after a run at the higher CC point. For both analyses carried out at ICL and at Syft, carryover was absent for all compounds (data not shown).

The use of liquid standards dissolved in methanol to build a CC for quantification of gaseous compounds is widely accepted, but it may be not perfectly accurate. The behavior of some of the compounds, for example in the interaction with the sorbent phase of the TD tubes, could present some differences, and the chemical standards used for the calibration may not be fully representative of the molecules measured in the breath. The use of gaseous standards, on the other hand, presents practical limitations that would have reduced the applicability of the method in both clinical practice and high-throughput analysis scenarios, negating one of the main advantages of the TD-SIFT-MS application. In general, the use of Biomonitoring TD tubes was not a perfect fit for all analytes. Fatty acids, especially acetic acid, are likely more suitably trapped on an alternative sorbent. The use of these tubes represents a compromise to extend the coverage of different compounds included in the method, ranging from C2 to C13.

## Conclusions

The combination of SIFT-MS and TD brings together the analysis speed and user-friendly features of SIFT-MS with the possibility to collect breath in a multicenter fashion given by TD tubes. This novel instrument has the potential to be easily employed in clinical settings for targeted analysis, using quick and completely automated analytical methods.

## Supporting Information Available

Calibration curves in different matrices (Figure S1); list of compounds included in the analytical method (Table S1); concentrations of each calibration curve point (Table S2); linearity, LOD, and LOQ obtained at Imperial College London and Syft Technologies (Table S3); accuracy and precision obtained at Imperial College London and Syft Technologies (Table S4) (PDF)
