# Supplementary material for: Combining Thermal Desorption with Selected Ion Flow Tube Mass Spectrometry for Analyses of Breath Volatile Organic Compounds
Source: Anal Chem. 2024 Jan 20;96(4):1397–401. doi: 10.1021/acs.analchem.3c04286 (PMC10831795; doi:10.1021/acs.analchem.3c04286)
Supplement: Supplementary file 1 — ac3c04286_si_001.pdf [file ac3c04286_si_001.pdf]

Supporting Information

Combining thermal desorption with selected ion flow tube mass spectrometry (TD-SIFT-MS) for analyses of breath volatile organic compounds.

Ilaria Belluomo[1], Sophia E Whitlock[2], Antonis Myridakis[1], Aaron G Parker[1], Valerio Converso[1], Mark J Perkins[3], Vaughan S Langford[2], Patrik Spanel[1,4], George B Hanna[1*].

[1]Department of Surgery and Cancer, Imperial College London, London W12 0HS, United Kingdom

[2]Syft Technologies Limited, 68 St Asaph St, Christchurch 8011, New Zealand.

[3]Element Lab Solutions, Wellbrook Court, Girton Road, Cambridge, CB3 0NA, United Kingdom.

[4]J. Heyrovský Institute of Physical Chemistry of the Czech Academy of Sciences, 182 23 Prague, Czech Republic.

* Email: g.hanna@imperial.ac.uk

TABLE OF CONTENTS

Table S1. List of compounds included in the analytical method.

Table S2. Concentrations of each calibration curve point.

Table S3. Linearity, LOD and LOQ obtained at Imperial College London and Syft Technologies.

Table S4. Accuracy and precision obtained at Imperial College London and Syft Technologies.

Figure S1. Calibration curves in different matrices.

Table S1. List of compounds included in the analytical method.

All the compounds included in the method, for quantification or monitoring purpose, are listed below with reagent ion, reaction rate, branching ratio and product ion. Expected breath concentrations are indicated for compounds included for breath quantification purpose (Low: < 20ppbv; Medium: > 20 ppbv but <100 ppbv; High: > 100ppbv).

Compound		Reagent Ion	Reaction Rate	Branching Ratio	Product Ion (mass, formula)	
Humidity	H_2O	NO^+	1.00E-10	100	48	$H_2O.NO^+$

Compound		Reagent Ion	Reaction Rate	Branching Ratio	Product Ion (mass, formula)	
1-Propanol	C_3H_8O	NO^+	2.30E-09	100	59	$C_3H_7O^+$
Ammonia	NH_3	H_3O^+	2.60E-09	100	18	NH_4^+
		H_3O^+	2.60E-09		36	$NH_4^+.H_2O$
Ethanol	C_2H_5OH	NO^+	1.20E-09	100	45	$C_2H_5O^+$
		NO^+	1.20E-09		63	$C_2H_5O^+.H_2O$
Methanol	CH_3OH	H_3O^+	2.70E-09	100	33	CH_5O^+
		H_3O^+	2.70E-09		51	$CH_3OH_2^+.H_2O$
		NO^+	1.00E-11	100	62	$NO^+.CH_3OH$

Compound		Reagent Ion	Reaction Rate	Branching Ratio	Product Ion (mass, formula)		Expected Breath Concentrations
Acetic Acid	CH_3COOH	NO^+	9.00E-10	100	90	$NO^+.CH_3COOH$	Medium
Acetone	C_3H_6O	NO^+	1.20E-09	100	88	$NO^+.C_3H_6O$	High
Benzaldehyde	C_7H_6O	NO^+	2.80E-09	100	105	$C_7H_5O^+$	Low

Butanal	C_4H_8O	NO^+	3.50E-09	100	71	$C_4H_7O^+$	Low
Butanoic Acid	$C_4H_8O_2$	NO^+	2.40E-09	41	118	$NO^+.C_3H_7COOH$	Low
Cyclohexane	C_6H_{12}	$O2^+$	1.30E-09	62	84	$C_6H_{12}^+$	Low
Decanal	$C_{10}H_{20}O$	H_3O^+	3.90E-09	97	157	$C_{10}H_{21}O^+$	Low
		H_3O^+	3.90E-09		175	$C_{10}H_{21}O^+.H_2O$	
		NO^+	3.30E-09	100	155	$C_{10}H_{19}O^+$	
Dodecane	$C_{12}H_{26}$	NO^+	2.00E-09	86	169	$C_{12}H_{25}^+$	Low
		O2+	2.00E-09	29	170	$C_{12}H_{26}^+$	
Hexanal	$C_6H_{12}O$	NO^+	2.50E-09	100	99	$C_6H_{11}O^+$	Low
Hexanoic Acid	$C_6H_{12}O_2$	NO^+	2.50E-09	54	146	$C_6H_{12}O_2.NO^+$	Low
Isoprene	C_5H_8	O2+	1.70E-09	45	67	$C_5H_7^+$	High
Nonanal	$C_9H_{18}O$	H_3O^+	2.50E-09	86	143	$C_9H_{19}O^+$	Low
		H_3O^+	2.50E-09		161	$C_9H_{19}O^+.H_2O$	
		NO^+	2.70E-09	100	141	$C_9H_{17}O^+$	
Nonanol	$C_9H_{20}O$	NO^+	2.30E-09	91	143	$C_9H_{19}O^+$	Low
Octanal	$C_8H_{16}O$	H_3O^+	3.80E-09	85	129	$C_8H_{17}O^+$	Low
		H_3O^+	3.80E-09		147	$C_8H_{17}O^+.2H_2O$	
		NO^+	3.00E-09	100	127	$C_8H_{15}O^+$	
Pentanoic Acid	$C_5H_{10}O_2$	NO^+	2.40E-09	41	132	$NO^+.C_4H_9COOH$	Low
Phenol	C_6H_6O	NO^+	2.00E-09	100	94	$C_6H_6O^+$	Low
Propanal	C_3H_6O	NO^+	2.50E-09	100	57	$C_3H_5O^+$	Low
		O2+	3.10E-09	50	57	$C_3H_5O^+$	

Propanoic Acid	$C_3H_6O_2$	NO^+	1.50E-09	69	104	$NO^+.C_2H_5COOH$	Low
Toluene	C_7H_8	NO^+	1.70E-09	100	92	$C_7H_8^+$	Low
Tridecane	$C_{13}H_{28}$	NO^+	2.00E-09	86	183	$C_{13}H_{27}^+$	Low
		O2+	2.00E-09	36	184	$C_{13}H_{28}^+$	
Undecanal	$C_{11}H_{22}O$	H_3O^+	3.00E-09	49	171	$C_{11}H_{22}O.H^+$	Low

Table S2. Concentrations of each calibration curve point.

	Cal 0	Cal 0.5	Cal 1	Cal 2.5	Cal 5	Cal 10	Cal 25	Cal 50	Cal 100
Acetone (ppbv)	0	10	20	50	100	200	500	1000	2000

Isoprene (ppbv)	0	2.5	5	12.5	25	50	125	250	500
Acetic Acid (ppbv)	0	1.25	2.5	6.25	12.5	25	62.5	125	250
All other VOCs (ppbv)	0	0.5	1	2.5	5	10	25	50	100

Table S3. Linearity, LOD and LOQ obtained at Imperial College London and Syft Technologies.

Imperial College London							
Compound	Matrix	Slope (mean ± SD)	Intercept (mean ± SD)	R^2 (mean ± SD)	Calibration Range	LOD (ppbv)	LOQ (ppbv)
Acetic Acid	MeOH	0.483 ± 0.014	1.662 ± 0.657	0.9974 ± 0.001	6.25-250 ppbv	13.634	45.447
	Breath	0.481 ± 0.014	3.118 ± 1.446	0.989 ± 0.004	12.5-250 ppbv	28.311	94.369

	Water	0.438 ± 0.096	1.719 ± 1.133	0.9945 ± 0.002	12.5-250 ppbv	19.94	66.47
Acetone	MeOH	1.515 ± 0.086	2.642 ± 2.709	0.9998 ± 0.0003	10-2000 ppbv	31.784	105.947
	Breath	1.262 ± 0.123	404.69 ± 25.894	0.9937 ± 0.002	n/a	n/a	n/a
	Water	1.089 ± 0.058	17.571 ± 3.799	0.9974 ± 0.001	50-2000 ppbv	107.355	357.850
Benzaldehyde	MeOH	6.59 ± 0.712	2.204 ± 0.321	0.9995 ± 0.0005	1-100 ppbv	2.249	7.497
	Breath	4.574 ± 0.16	6.958 ± 1.612	0.9976 ± 0.002	2.5-100 ppbv	5.083	16.944
	Water	3.38 ± 0.203	10.661 ± 1.519	0.988 ± 0.006	5-100 ppbv	11.716	39.054
Butanal	MeOH	3.158 ± 0.126	0.487 ± 0.561	0.9997 ± 0.0003	2.5-100 ppbv	1.908	6.361
	Breath	3.256 ± 0.18	0.875 ± 0.836	0.9998 ± 0.0002	2.5-100 ppbv	1.514	5.047
	Water	3.003 ± 0.166	0.575 ± 0.449	0.9996 ± 0.0001	1-100 ppbv	2.040	6.800
Butanoic Acid	MeOH	4.592 ± 0.137	3.724 ± 1.232	0.9988 ± 0.001	2.5-100 ppbv	3.598	11.992
	Breath	4.658 ± 0.325	7.329 ± 2.495	0.9987 ± 0.001	5-100 ppbv	3.667	12.224
	Water	4.49 ± 0.352	5.103 ± 3.462	0.9984 ± 0.001	2.5-100 ppbv	4.324	14.415
Cyclohexane	MeOH	10.395 ± 0.168	2.555 ± 2.218	0.9995 ± 0.0004	1-100 ppbv	2.235	7.450
	Breath	10.643 ± 0.346	4.297 ± 2.707	0.9998 ± 0.0001	1-100 ppbv	1.352	4.507
	Water	10.02 ± 0.03	5.35 ± 0.694	0.9992 ± 0.0001	2.5-100 ppbv	3.071	10.236
Decanal	MeOH	8.505 ± 0.35	2.3 ± 1.93	0.9993 ± 0.0004	2.5-100 ppbv	2.703	9.010
	Breath	8.896 ± 0.205	6.852 ± 3.055	0.9992 ± 0	2.5-100 ppbv	2.987	9.957
	Water	7.991 ± 0.403	13.909 ± 3.473	0.997 ± 0.0003	5-100 ppbv	5.927	19.757
Dodecane	MeOH	9.905 ± 0.512	10.253 ± 2.821	0.999 ± 0.001	1-100 ppbv	2.256	7.520
	Breath	9.664 ± 0.416	9.495 ± 0.566	0.9992 ± 0.001	2.5-100 ppbv	2.558	8.526
	Water	9.187 ± 0.04	4.78 ± 0.314	0.9985 ± 0.001	2.5-100 ppbv	4.132	13.775
Hexanal	MeOH	9.856 ± 0.31	5.495 ± 4.505	0.9992 ± 0.001	2.5-100 ppbv	2.544	8.479
	Breath	9.625 ± 0.176	6.857 ± 6.191	0.9995 ± 0.0002	2.5-100 ppbv	2.200	7.332
	Water	8.872 ± 0.061	2.549 ± 2.324	0.9997 ± 0.0002	1-100 ppbv	1.893	6.310
Hexanoic Acid	MeOH	6.158 ± 0.4	7.643 ± 1.533	0.9987 ± 0.001	2.5-100 ppbv	3.711	12.370
	Breath	5.791 ± 0.177	9.735 ± 8.331	0.9983 ± 0.002	2.5-100 ppbv	3.458	11.525
	Water	5.508 ± 0.05	10.659 ± 6.309	0.9984 ± 0.001	2.5-100 ppbv	3.796	12.654
Isoprene	MeOH	1.815 ± 0.047	5.156 ± 0.19	0.999 ± 0.0008	12.5-500 ppbv	15.606	52.019
	Breath	1.778 ± 0.109	10.823 ± 1.139	0.994 ± 0.001	n/a	n/a	n/a
	Water	1.664 ± 0.081	7.832 ± 2.945	0.9984 ± 0.0005	12.5-500 ppbv	21.776	72.587
Nonanal	MeOH	10.472 ± 0.5	2.174 ± 0.677	0.9994 ± 0.0005	2.5-100 ppbv	2.506	8.354
	Breath	9.818 ± 0.702	6.901 ± 0.698	0.9991 ± 0.001	2.5-100 ppbv	3.099	10.330
	Water	8.792 ± 0.38	19.416 ± 1.286	0.9935 ± 0.001	2.5-100 ppbv	8.720	29.066
Nonanol	MeOH	11.172 ± 0.347	4.309 ± 4.262	0.9992 ± 0.001	2.5-100 ppbv	2.644	8.812
	Breath	11.164 ± 0.535	3.338 ± 3.197	0.9996 ± 0.0005	2.5-100 ppbv	1.932	6.440
	Water	10.867 ± 0.036	4.343 ± 1.066	0.9991 ± 0.0001	5-100 ppbv	3.250	10.833
Octanal	MeOH	4.808 ± 0.095	0.804 ± 0.533	0.999 ± 0.001	2.5-100 ppbv	3.121	10.403
	Breath	5.045 ± 0.007	1.44 ± 0.329	0.9994 ± 0.001	1-100 ppbv	2.427	8.091
	Water	4.455 ± 0.015	2.844 ± 0.452	0.9991 ± 0.001	2.5-100 ppbv	3.145	10.484
Pentanoic Acid	MeOH	6.282 ± 0.616	5.291 ± 1.977	0.999 ± 0.001	2.5-100 ppbv	3.420	11.400
	Breath	5.558 ± 0.307	4.407 ± 2.763	0.9992 ± 0.001	2.5-100 ppbv	2.827	9.422
	Water	5.364 ± 0.208	7.051 ± 2.114	0.9987 ± 0.001	2.5-100 ppbv	3.673	12.245
Phenol	MeOH	4.241 ± 0.113	1.571 ± 0.655	0.9994 ± 0.0005	2.5-100 ppbv	2.589	8.629
	Breath	4.304 ± 0.07	0.685 ± 0.058	0.9999 ± 0.0001	1-100 ppbv	1.068	3.559
	Water	4.144 ± 0.008	0.267 ± 0.253	0.9998 ± 0	1-100 ppbv	1.542	5.142
Propanal	MeOH	1.935 ± 0.06	0.14 ± 0.125	0.9996 ± 0.0003	2.5-100 ppbv	2.107	7.022

	Breath	1.725 ± 0.099	0.44 ± 0.065	0.9984 ± 0.0005	2.5-100 ppbv	4.329	14.429
	Water	1.536 ± 0.396	0.601 ± 0.542	0.9987 ± 0.001	2.5-100 ppbv	3.572	11.908
Propanoic Acid	MeOH	2.955 ± 0.071	3.211 ± 0.248	0.9986 ± 0.0004	2.5-100 ppbv	3.959	13.197
	Breath	3.025 ± 0.183	12.633 ± 1.324	0.9942 ± 0	5-100 ppbv	8.296	27.652
	Water	2.872 ± 0.384	2.856 ± 0.826	0.9981 ± 0.0004	2.5-100 ppbv	4.757	15.855
Toluene	MeOH	4.65 ± 0.155	0.572 ± 0.6	0.9995 ± 0.0006	1-100 ppbv	2.130	7.099
	Breath	4.894 ± 0.133	0.584 ± 0.621	0.9996 ± 0.0001	1-100 ppbv	2.191	7.303
	Water	4.654 ± 0.038	0.449 ± 0.011	0.9998 ± 0.0001	1-100 ppbv	1.786	5.952
Tridecane	MeOH	11.146 ± 0.182	8.39 ± 5.495	0.9985 ± 0.002	2.5-100 ppbv	3.303	11.010
	Breath	11.674 ± 0.725	7.68 ± 4.512	0.9991 ± 0.001	5-100 ppbv	2.972	9.906
	Water	11.186 ± 0.18	7.259 ± 3.758	0.999 ± 0.0003	5-100 ppbv	3.435	11.449
Undecanal	MeOH	25.225 ± 2.222	8.472 ± 11.459	0.9993 ± 0.001	1-100 ppbv	2.577	8.590
	Breath	23.812 ± 0.629	9.307 ± 3.642	0.9996 ± 0.0001	2.5-100 ppbv	2.176	7.253
	Water	21.828 ± 0.811	15.132 ± 5.856	0.997 ± 0.002	5-100 ppbv	5.741	19.138

Syft Technologies

Compound	Matrix	Slope (mean ± SD)	Intercept (mean ± SD)	R^2 (mean ± SD)	Range	LOD (ppbv)	LOQ (ppbv)
Acetic Acid	MeOH	0.205 ± 0.007	0.541 ± 0.203	0.9899 ± 0.016	12.5-100 ppbv	20.231	67.436
	Breath	0.183 ± 0.004	0.506 ± 0.12	0.996 ± 0.004	12.5-100 ppbv	16.183	53.945
	Water	0.23 ± 0.003	0.683 ± 0.662	0.9959 ± 0.004	25-100 ppbv	16.093	53.645
Acetone	MeOH	0.746 ± 0.021	4.175 ± 1.737	0.9999 ± 0.0001	50-100 ppbv	26.112	87.040
	Breath	0.795 ± 0.0139	32.75 ± 11.053	0.9969 ± 0.0008	n/a	n/a	n/a
	Water	0.757 ± 0.002	1.567 ± 1.141	0.9999 ± 0.0001	10-100 ppbv	27.404	91.346
Benzaldehyde	MeOH	2.347 ± 0.03	0.38 ± 0.176	0.9999 ± 0.0001	1-100 ppbv	1.025	3.418
	Breath	2.38 ± 0.02	1.685 ± 0.237	0.9991 ± 0.0001	2.5-100 ppbv	3.239	10.797
	Water	2.323 ± 0.004	1.61 ± 0.061	0.9997 ± 0	1-100 ppbv	1.921	6.404
Butanal	MeOH	1.806 ± 0.03	0.979 ± 0.029	0.9993 ± 0.001	2.5-100 ppbv	2.658	8.861
	Breath	1.733 ± 0.01	1.202 ± 0.031	0.9991 ± 0.0003	2.5-100 ppbv	3.198	10.660
	Water	1.912 ± 0.012	0.5 ± 0.016	0.9996 ± 0.0001	2.5-100 ppbv	2.216	7.387
Butanoic Acid	MeOH	1.426 ± 0.058	2.229 ± 0.292	0.9954 ± 0.004	2.5-100 ppbv	6.826	22.752
	Breath	1.319 ± 0.025	1.386 ± 0.235	0.9978 ± 0.001	2.5-100 ppbv	5.079	16.930
	Water	1.621 ± 0.011	0.797 ± 0.409	0.9973 ± 0.003	2.5-100 ppbv	5.265	17.550
Cyclohexane	MeOH	5.227 ± 0.027	0.619 ± 0.101	0.9999 ± 0.0001	1-100 ppbv	0.964	3.215
	Breath	5.165 ± 0.029	2.09 ± 0.497	0.9999 ± 0	2.5-100 ppbv	1.260	4.201
	Water	5.369 ± 0.057	3.305 ± 0.345	0.9998 ± 0.0001	1-100 ppbv	1.555	5.185
Decanal	MeOH	3.564 ± 0.046	1.698 ± 0.269	0.9989 ± 0.001	5-100 ppbv	3.538	11.794
	Breath	3.972 ± 0.223	2.884 ± 1.994	0.9988 ± 0.002	1-100 ppbv	3.125	10.416
	Water	4.116 ± 0.007	0.839 ± 0.718	0.9984 ± 0.002	2.5-100 ppbv	3.960	13.199
Dodecane	MeOH	4.834 ± 0.073	2.205 ± 0.741	0.9995 ± 0.0002	1-100 ppbv	2.630	8.766
	Breath	4.679 ± 0.017	0.353 ± 0.063	0.9996 ± 0.0004	2.5-100 ppbv	2.182	7.272
	Water	5.28 ± 0.05	1.019 ± 0.866	0.9993 ± 0.0002	2.5-100 ppbv	2.987	9.957
Hexanal	MeOH	3.365 ± 0.03	1.344 ± 0.287	0.9994 ± 0.001	2.5-100 ppbv	2.393	7.978
	Breath	3.366 ± 0.054	2.852 ± 1.008	0.9989 ± 0.0001	2.5-100 ppbv	3.540	11.799
	Water	3.639 ± 0.011	2.773 ± 3.652	0.9996 ± 0.0004	1-100 ppbv	1.668	5.559
Hexanoic Acid	MeOH	2.255 ± 0.045	5.109 ± 0.908	0.9892 ± 0.011	5-100 ppbv	10.345	34.482
	Breath	2.234 ± 0.063	4.585 ± 0.262	0.9951 ± 0.001	5-100 ppbv	7.480	24.933
	Water	2.694 ± 0.02	3.972 ± 1.234	0.9952 ± 0.004	5-100 ppbv	6.993	23.309
Isoprene	MeOH	1.054 ± 0.041	4.362 ± 0.438	0.9989 ± 0.0002	12.5-500 ppbv	17.874	59.580

	Breath	1.029 ± 0.003	9.391 ± 3.677	0.977 ± 0.0003	n/a	n/a	n/a
	Water	0.522 ± 0.011	0.345 ± 0.325	0.999 ± 0.001	25-100 ppbv	15.891	52.970
Nonanal	MeOH	3.587 ± 0.184	3.909 ± 1.926	0.9953 ± 0.003	5-100 ppbv	6.947	23.157
	Breath	4.151 ± 0.226	1.592 ± 1.836	0.9989 ± 0	1-100 ppbv	2.217	7.391
	Water	4.064 ± 0.188	3.153 ± 2.506	0.9961 ± 0.004	5-100 ppbv	6.319	21.062
Nonanol	MeOH	4.937 ± 0.061	0.791 ± 0.179	0.9999 ± 0.0001	0.5-100 ppbv	0.814	2.712
	Breath	4.852 ± 0.001	1.649 ± 0.265	0.9999 ± 0	1-100 ppbv	0.922	3.074
	Water	5.611 ± 0.047	0.992 ± 0.036	0.9994 ± 0.0002	2.5-100 ppbv	2.758	9.194
Octanal	MeOH	1.896 ± 0.03	0.221 ± 0.155	0.9997 ± 0.0003	1-100 ppbv	1.695	5.650
	Breath	1.937 ± 0.046	1.264 ± 0.561	0.9989 ± 0.0005	2.5-100 ppbv	3.584	11.946
	Water	2.032 ± 0.061	0.818 ± 0.471	0.9998 ± 0.0001	2.5-100 ppbv	1.810	6.034
Pentanoic Acid	MeOH	1.923 ± 0.057	3.71 ± 0.539	0.9936 ± 0.005	5-100 ppbv	8.230	27.432
	Breath	1.813 ± 0.065	2.568 ± 0.783	0.9969 ± 0.0001	2.5-100 ppbv	6.092	20.305
	Water	2.203 ± 0.011	1.465 ± 0.298	0.9967 ± 0.003	2.5-100 ppbv	5.802	19.339
Phenol	MeOH	1.089 ± 0.005	0.339 ± 0.073	0.9999 ± 0.0001	2.5-100 ppbv	1.027	3.423
	Breath	1.075 ± 0.004	0.689 ± 0.324	0.9993 ± 0.0003	1-100 ppbv	2.833	9.444
	Water	1.045 ± 0.002	0.117 ± 0.086	0.9999 ± 0.0001	2.5-100 ppbv	1.434	4.779
Propanal	MeOH	0.965 ± 0.005	0.39 ± 0.103	0.9996 ± 0.0003	1-100 ppbv	2.022	6.739
	Breath	0.607 ± 0.017	0.467 ± 0.484	0.9991 ± 0.0001	2.5-100 ppbv	3.186	10.620
	Water	0.865 ± 0.03	0.205 ± 0.054	0.9995 ± 0.0001	2.5-100 ppbv	2.576	8.587
Propanoic Acid	MeOH	0.886 ± 0.045	0.939 ± 0.215	0.9952 ± 0.007	2.5-100 ppbv	5.992	19.973
	Breath	0.809 ± 0.018	0.831 ± 0.098	0.9983 ± 0.002	2.5-100 ppbv	4.199	13.995
	Water	0.986 ± 0.007	0.367 ± 0.264	0.9986 ± 0.002	2.5-100 ppbv	3.625	12.082
Toluene	MeOH	2.071 ± 0.008	0.546 ± 0.343	0.9998 ± 0.0001	2.5-100 ppbv	1.433	4.778
	Breath	2.087 ± 0.005	1.474 ± 0.308	0.9992 ± 0.0001	2.5-100 ppbv	3.080	10.267
	Water	2.099 ± 0.002	0.789 ± 0.562	0.9999 ± 0	1-100 ppbv	1.173	3.910
Tridecane	MeOH	5.101 ± 0.103	2.015 ± 1.612	0.999 ± 0.001	2.5-100 ppbv	3.171	10.570
	Breath	4.993 ± 0.025	0.828 ± 0.993	0.9999 ± 0.0001	1-100 ppbv	1.344	4.481
	Water	5.065 ± 0.027	5.708 ± 0.486	0.9988 ± 0.0005	2.5-100 ppbv	3.785	12.618
Undecanal	MeOH	12.487 ± 0.344	2.025 ± 1.87	0.9999 ± 0.0001	1-100 ppbv	1.023	3.411
	Breath	12.45 ± 0.092	7.074 ± 1.3	0.9997 ± 0.0001	2.5-100 ppbv	1.837	6.124
	Water	14.221 ± 0.157	1.988 ± 2.653	0.9996 ± 0.0004	1-100 ppbv	1.929	6.431

Table S4. Accuracy and precision obtained at Imperial College London and Syft Technologies.

Imperial College London						
		Standard addition (ppbv)	Measured Level (mean ± SD)	Accuracy %	CV% intra-day	CV% inter-day
Acetic Acid	Cal 6.25	6.25	8.747 ± 1.298	139.95	14.38	14.84
	Cal 12.5	12.5	12.169 ± 1.313	97.35	10.03	10.79
	Cal 25	25	23.47 ± 2.507	93.88	9.61	10.68
	Cal 125	125	138.239 ± 11.598	110.59	7.43	8.39
	Cal 250	250	309.579 ± 20.849	123.83	6.93	6.73
Acetone	Cal 50	50	45.45 ± 3.151	90.90	4.74	6.93
	Cal 100	100	109.515 ± 4.902	109.52	4.28	4.48
	Cal 200	200	222.526 ± 11.456	111.26	5.07	5.15
	Cal 1000	1000	1085.943 ± 64.721	108.59	5.00	5.96
	Cal 2000	2000	2185.404 ± 143.709	109.27	6.61	6.58
Benzaldehyde	Cal 2.5	2.5	2.788 ± 0.411	111.54	11.40	14.72
	Cal 5	5	5.532 ± 0.284	110.63	3.65	5.14
	Cal 10	10	11.263 ± 0.632	112.63	4.68	5.61
	Cal 50	50	53.267 ± 2.737	106.53	4.36	5.14
	Cal 100	100	109.833 ± 6.027	109.83	4.86	5.49
Butanal	Cal 2.5	2.5	2.711 ± 0.153	108.42	4.26	5.64
	Cal 5	5	5.291 ± 0.406	105.82	3.65	7.68
	Cal 10	10	10.722 ± 0.68	107.22	5.11	6.35
	Cal 50	50	56.119 ± 2.881	112.24	4.96	5.13
	Cal 100	100	115.11 ± 6.195	115.11	5.27	5.38
Butanoic Acid	Cal 2.5	2.5	2.759 ± 0.319	110.34	10.89	11.58
	Cal 5	5	4.774 ± 0.446	95.49	9.12	9.35
	Cal 10	10	10.114 ± 0.696	101.14	6.50	6.88
	Cal 50	50	54.033 ± 3.189	108.07	5.80	5.90
	Cal 100	100	111.197 ± 5.119	111.20	4.44	4.60
Cyclohexane	Cal 2.5	2.5	2.374 ± 0.229	94.98	9.73	9.64
	Cal 5	5	5.356 ± 0.216	107.12	3.55	4.04
	Cal 10	10	11.011 ± 0.572	110.11	3.81	5.19
	Cal 50	50	54.157 ± 2.383	108.31	4.28	4.40
	Cal 100	100	108.201 ± 4.861	108.20	4.29	4.49
Decanal	Cal 2.5	2.5	3.013 ± 0.389	120.53	9.84	12.91
	Cal 5	5	5.735 ± 0.301	114.69	4.31	5.26
	Cal 10	10	11.13 ± 0.722	111.30	4.63	6.48
	Cal 50	50	53.798 ± 2.375	107.60	4.47	4.42
	Cal 100	100	112.374 ± 6.345	112.37	5.48	5.65
Dodecane	Cal 2.5	2.5	2.295 ± 0.343	91.79	15.76	14.93
	Cal 5	5	5.42 ± 0.416	108.40	4.47	7.67

	Cal 10	10	11.355 ± 0.883	113.55	5.07	7.77
	Cal 50	50	54.557 ± 2.252	109.11	4.02	4.13
	Cal 100	100	106.842 ± 4.548	106.84	4.27	4.26
Hexanal	Cal 2.5	2.5	2.777 ± 0.166	111.09	6.25	5.99
	Cal 5	5	5.362 ± 0.218	107.24	3.61	4.07
	Cal 10	10	10.97 ± 0.555	109.70	4.77	5.06
	Cal 50	50	54.627 ± 2.551	109.25	4.76	4.67
	Cal 100	100	113.208 ± 5.562	113.21	4.63	4.91
Hexanoic Acid	Cal 2.5	2.5	2.379 ± 0.378	95.16	15.11	15.89
	Cal 5	5	4.811 ± 0.464	96.23	9.95	9.63
	Cal 10	10	10.21 ± 0.7	102.10	6.14	6.85
	Cal 50	50	55.644 ± 3.441	111.29	6.51	6.18
	Cal 100	100	115.377 ± 5.517	115.38	4.66	4.78
Isoprene	Cal 12.5	12.5	12.013 ± 1.055	96.10	8.03	8.78
	Cal 25	25	23.868 ± 1.526	95.47	4.48	6.39
	Cal 50	50	48.981 ± 3.634	97.96	4.99	7.42
	Cal 250	250	255.017 ± 18.448	102.01	5.11	7.23
	Cal 500	500	515.113 ± 39.711	103.02	6.45	7.71
Nonanal	Cal 2.5	2.5	2.818 ± 0.283	112.73	8.21	10.03
	Cal 5	5	5.782 ± 0.293	115.64	4.49	5.06
	Cal 10	10	11.324 ± 0.705	113.24	4.62	6.22
	Cal 50	50	53.974 ± 2.522	107.95	4.65	4.67
	Cal 100	100	111.711 ± 6.331	111.71	5.43	5.67
Nonanol	Cal 2.5	2.5	2.303 ± 0.167	92.13	6.29	7.27
	Cal 5	5	5.587 ± 0.332	111.74	4.67	5.94
	Cal 10	10	11.427 ± 0.625	114.27	4.57	5.47
	Cal 50	50	55.611 ± 2.531	111.22	4.67	4.55
	Cal 100	100	109.77 ± 5.509	109.77	4.59	5.02
Octanal	Cal 2.5	2.5	3.163 ± 0.38	126.51	9.08	12.01
	Cal 5	5	5.789 ± 0.252	115.79	4.23	4.35
	Cal 10	10	11.227 ± 0.628	112.27	4.88	5.59
	Cal 50	50	53.458 ± 2.469	106.92	4.56	4.62
	Cal 100	100	111.912 ± 7.013	111.91	6.07	6.27
Pentanoic Acid	Cal 2.5	2.5	2.712 ± 0.396	108.49	14.58	14.61
	Cal 5	5	5.068 ± 0.5	101.36	8.50	9.87
	Cal 10	10	10.631 ± 0.806	106.31	6.30	7.58
	Cal 50	50	55.795 ± 3.04	111.59	5.71	5.45
	Cal 100	100	113.552 ± 5.355	113.55	4.66	4.72
Phenol	Cal 2.5	2.5	2.656 ± 0.146	106.24	5.17	5.51
	Cal 5	5	5.527 ± 0.255	110.54	4.23	4.62
	Cal 10	10	11.163 ± 0.661	111.63	5.95	5.93
	Cal 50	50	55.287 ± 2.706	110.57	4.58	4.90
	Cal 100	100	112.5 ± 4.876	112.50	4.16	4.33

Propanal	Cal 2.5	2.5	2.848 ± 0.387	113.91	6.41	13.60
	Cal 5	5	5.463 ± 0.195	109.26	3.25	3.57
	Cal 10	10	10.693 ± 0.589	106.93	5.55	5.51
	Cal 50	50	55.583 ± 3.121	111.17	4.88	5.62
	Cal 100	100	112.346 ± 6.597	112.35	5.90	5.87
Propanoic Acid	Cal 2.5	2.5	3.24 ± 0.316	129.59	10.10	9.75
	Cal 5	5	4.696 ± 0.467	93.92	10.09	9.95
	Cal 10	10	9.617 ± 0.756	96.17	6.75	7.86
	Cal 50	50	53.92 ± 3.48	107.84	6.04	6.45
	Cal 100	100	112.871 ± 5.84	112.87	4.66	5.17
Toluene	Cal 2.5	2.5	2.544 ± 0.128	101.77	5.07	5.02
	Cal 5	5	5.424 ± 0.283	108.49	4.91	5.21
	Cal 10	10	10.913 ± 0.6	109.13	4.96	5.50
	Cal 50	50	55.233 ± 2.78	110.47	4.57	5.03
	Cal 100	100	112.905 ± 5.671	112.90	4.87	5.02
Tridecane	Cal 2.5	2.5	2.381 ± 0.244	95.22	9.18	10.24
	Cal 5	5	5.258 ± 0.427	105.16	4.58	8.11
	Cal 10	10	11.384 ± 0.761	113.84	4.24	6.69
	Cal 50	50	54.625 ± 2.487	109.25	4.25	4.55
	Cal 100	100	105.425 ± 3.973	105.43	3.35	3.77
Undecanal	Cal 2.5	2.5	2.79 ± 0.352	111.60	7.51	12.63
	Cal 5	5	5.6 ± 0.34	112.00	5.72	6.07
	Cal 10	10	11.08 ± 0.674	110.80	5.41	6.08
	Cal 50	50	55.13 ± 2.205	110.26	3.99	4.00
	Cal 100	100	109.68 ± 5.357	109.68	4.43	4.88

Syft Technologies						
		Standard addition (ppbv)	Measured Level (mean ± SD)	Accuracy %	CV% intra-day	CV% inter-day
Acetic Acid	Cal 6.25	6.25	14.441 ± 4.992	231.048	34.571	
	Cal 12.5	12.5	13.503 ± 1.513	108.023	10.569	11.207
	Cal 25	25	26.908 ± 1.825	107.634	6.249	6.782
	Cal 125	125	127.123 ± 4.823	101.698	3.282	3.794
	Cal 250	250	260.096 ± 5.556	104.038	1.937	2.136
Acetone	Cal 50	50	57.342 ± 3.391	114.684	5.914	
	Cal 100	100	102.537 ± 1.571	102.537	1.169	1.532
	Cal 200	200	198.572 ± 3.672	99.286	1.345	1.849
	Cal 1000	1000	980.727 ± 15.741	98.073	0.812	1.605
	Cal 2000	2000	1987.679 ± 39.819	99.384	0.801	2.003
Benzaldehyde	Cal 2.5	2.5	3.414 ± 0.21	136.566	6.161	
	Cal 5	5	5.059 ± 0.172	101.173	2.896	3.405
	Cal 10	10	9.886 ± 0.289	98.862	2.160	2.923

	Cal 50	50	48.969 ± 1.026	97.937	0.922	2.096
	Cal 100	100	98.306 ± 1.987	98.306	1.133	2.022
Butanal	Cal 2.5	2.5	3.511 ± 0.463	140.435	13.201	
	Cal 5	5	5.221 ± 0.153	104.427	2.604	2.935
	Cal 10	10	9.952 ± 0.236	99.522	2.043	2.375
	Cal 50	50	50.081 ± 0.963	100.161	1.300	1.923
	Cal 100	100	102.148 ± 1.006	102.148	0.892	0.985
Butanoic Acid	Cal 2.5	2.5	3.29 ± 0.231	131.593	7.015	
	Cal 5	5	6.176 ± 0.447	123.519	7.369	7.234
	Cal 10	10	10.931 ± 0.899	109.314	7.676	8.226
	Cal 50	50	52.074 ± 1.851	104.149	3.368	3.554
	Cal 100	100	106.427 ± 2.283	106.427	1.165	2.145
Cyclohexane	Cal 2.5	2.5	3.246 ± 0.412	129.822	12.700	
	Cal 5	5	4.955 ± 0.245	99.110	3.006	4.938
	Cal 10	10	9.886 ± 0.528	98.864	2.690	5.342
	Cal 50	50	49.6 ± 1.581	99.200	1.299	3.187
	Cal 100	100	100.294 ± 1.865	100.294	1.101	1.859
Decanal	Cal 2.5	2.5	4.15 ± 1.27	166.002	30.608	
	Cal 5	5	4.833 ± 0.253	96.665	4.554	5.239
	Cal 10	10	10.129 ± 0.535	101.290	3.990	5.286
	Cal 50	50	53.306 ± 2.134	106.611	3.142	4.003
	Cal 100	100	104.322 ± 4.748	104.322	4.465	4.551
Dodecane	Cal 2.5	2.5	2.657 ± 0.406	106.276	15.280	
	Cal 5	5	4.655 ± 0.431	93.106	8.227	9.250
	Cal 10	10	10.276 ± 0.629	102.764	4.156	6.119
	Cal 50	50	51.527 ± 2.476	103.054	3.069	4.806
	Cal 100	100	101.428 ± 2.699	101.428	1.653	2.661
Hexanal	Cal 2.5	2.5	3.33 ± 0.407	133.205	12.215	
	Cal 5	5	5.458 ± 0.24	109.162	2.631	4.392
	Cal 10	10	10.402 ± 0.324	104.021	1.701	3.112
	Cal 50	50	51.271 ± 1.207	102.543	1.810	2.354
	Cal 100	100	104.955 ± 1.791	104.955	1.652	1.706
Hexanoic Acid	Cal 2.5	2.5	4.609 ± 1.014	184.359	21.990	
	Cal 5	5	6.505 ± 0.602	130.101	6.266	9.258
	Cal 10	10	11.473 ± 0.977	114.728	5.429	8.517
	Cal 50	50	55.571 ± 3.345	111.143	5.353	6.020
	Cal 100	100	119.637 ± 3.229	119.637	2.542	2.699
Isoprene	Cal 12.5	12.5	16.345 ± 1.43	130.760	8.748	
	Cal 25	25	26.275 ± 1.064	105.099	2.266	4.050
	Cal 50	50	47.649 ± 1.358	95.299	1.301	2.850
	Cal 250	250	232.769 ± 8.346	93.107	0.798	3.586
	Cal 500	500	491.943 ± 16.08	98.389	0.908	3.269
Nonanal	Cal 2.5	2.5	3.415 ± 0.716	136.594	20.977	

	Cal 5	5	4.884 ± 0.353	97.686	6.825	7.225
	Cal 10	10	10.436 ± 0.438	104.361	2.945	4.195
	Cal 50	50	53.322 ± 1.71	106.644	2.549	3.207
	Cal 100	100	98.915 ± 6.249	98.915	6.681	6.318
Nonanol	Cal 2.5	2.5	2.66 ± 0.237	106.403	8.924	
	Cal 5	5	5.208 ± 0.196	104.169	3.329	3.759
	Cal 10	10	10.252 ± 0.254	102.524	2.042	2.477
	Cal 50	50	51.031 ± 1.053	102.062	1.340	2.063
	Cal 100	100	102.321 ± 1.8	102.321	1.339	1.759
Octanal	Cal 2.5	2.5	4.007 ± 0.601	160.291	15.008	
	Cal 5	5	4.989 ± 0.189	99.777	3.751	3.788
	Cal 10	10	9.853 ± 0.319	98.533	2.654	3.241
	Cal 50	50	50.116 ± 1.221	100.232	1.512	2.436
	Cal 100	100	103.139 ± 2.371	103.139	1.864	2.299
Pentanoic Acid	Cal 2.5	2.5	3.993 ± 0.502	159.708	12.570	
	Cal 5	5	6.607 ± 0.487	132.149	4.823	7.369
	Cal 10	10	11.208 ± 0.775	112.080	5.660	6.919
	Cal 50	50	53.612 ± 2.444	107.225	4.038	4.559
	Cal 100	100	111.14 ± 2.233	111.140	1.613	2.009
Phenol	Cal 2.5	2.5	2.927 ± 0.06	117.084	2.046	
	Cal 5	5	5.072 ± 0.243	101.438	4.222	4.784
	Cal 10	10	9.847 ± 0.269	98.468	2.740	2.732
	Cal 50	50	49.622 ± 0.892	99.244	0.847	1.797
	Cal 100	100	100.95 ± 1.46	100.950	0.919	1.446
Propanal	Cal 2.5	2.5	3.494 ± 0.768	139.753	21.988	
	Cal 5	5	5.056 ± 0.185	101.125	3.516	3.668
	Cal 10	10	9.817 ± 0.327	98.168	2.870	3.336
	Cal 50	50	49.587 ± 0.931	99.175	1.368	1.877
	Cal 100	100	100.76 ± 1.167	100.760	1.095	1.158
Propanoic Acid	Cal 2.5	2.5	4.131 ± 0.43	165.227	10.404	
	Cal 5	5	5.691 ± 0.442	113.827	7.564	7.760
	Cal 10	10	10.501 ± 0.672	105.015	5.306	6.399
	Cal 50	50	51.084 ± 1.605	102.167	2.704	3.143
	Cal 100	100	103.266 ± 2.169	103.266	1.725	2.101
Toluene	Cal 2.5	2.5	2.615 ± 0.096	104.613	3.661	
	Cal 5	5	4.933 ± 0.183	98.659	2.194	3.719
	Cal 10	10	9.583 ± 0.305	95.830	2.145	3.181
	Cal 50	50	48.224 ± 1.264	96.448	0.990	2.621
	Cal 100	100	100.055 ± 1.723	100.055	1.068	1.722
Tridecane	Cal 2.5	2.5	1.942 ± 0.154	77.677	7.943	
	Cal 5	5	4.997 ± 0.495	99.937	8.301	9.907
	Cal 10	10	10.28 ± 0.978	102.800	6.849	9.511
	Cal 50	50	51.463 ± 2.043	102.927	2.521	3.969

	Cal 100	100	101.133 ± 2.914	101.133	1.362	2.881
Undecanal	Cal 2.5	2.5	2.626 ± 0.132	105.022	5.034	
	Cal 5	5	5.041 ± 0.231	100.817	4.478	4.585
	Cal 10	10	10.063 ± 0.417	100.632	2.684	4.146
	Cal 50	50	51.459 ± 1.476	102.918	1.667	2.868
	Cal 100	100	101.982 ± 2.268	101.982	1.382	2.223

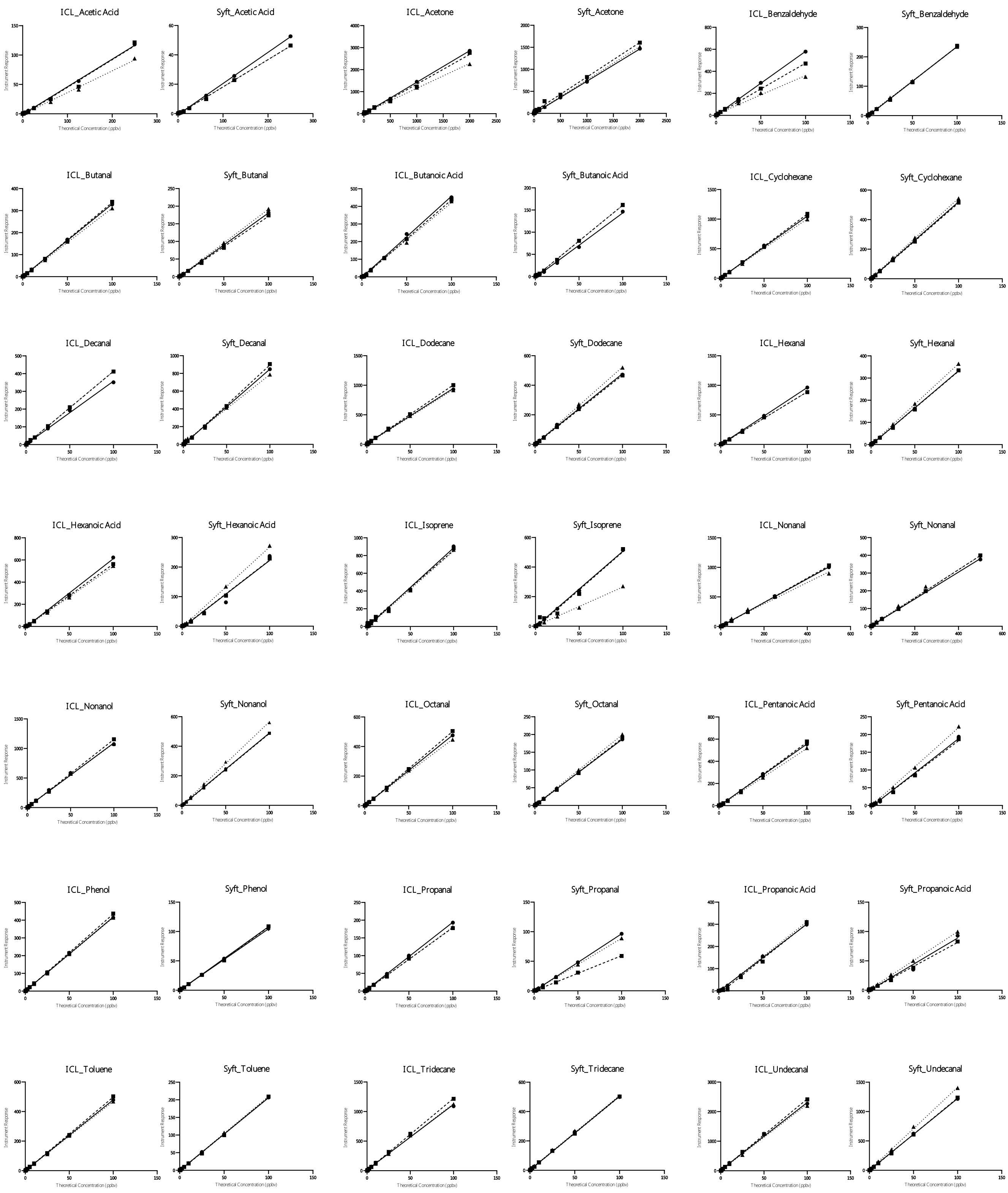

Figure S1: Three calibration curves made each one in a different biological matrix (standards in methanol, solid line; standards in water, dotted line; standards in breath from healthy volunteers, dashed line) were analyzed at the two laboratories where the method development and validation was carried out (ICL: Imperial College London, London, UK; Syft: Syft technologies, Christchurch, New Zealand) for each compound included in the analytical method. The overlap between the three

indicate similar effect of the three different biological matrices on the quantification of the compounds.
